# EccC3 is essential for pathogenesis of rough *Mycobacterium abscessus* in zebrafish

**DOI:** 10.1128/spectrum.01949-25

**Published:** 2025-09-29

**Authors:** Yara Tasrini, Wassim Daher, Laurent Kremer

**Affiliations:** 1 Centre National de la Recherche Scientifique UMR 9004, Institut de Recherche en Infectiologie de Montpellier (IRIM), Université de Montpellier 27037, Montpellier, France; 2 INSERM, IRIM 131821 , Montpellier, France; CNRS-University of Toulouse, Toulouse, France

**Keywords:** *Mycobacterium abscessus*, ESX-3, secretion system, pathogenesis, infection, zebrafish

## Abstract

*Mycobacterium abscessus* (*Mab*) is an emerging human pathogen causing severe lung infections in cystic fibrosis patients and displays either a smooth (S) or a rough (R) morphotype, the latter being associated with a strong inflammatory response. In contrast to most other mycobacteria, it possesses only two ESX secretion systems, ESX-3 and ESX-4. Recent work showed that infection of *Mab* S lacking the *eccC3*-encoding ATPase component of ESX-3 in mice was associated with reduced bacterial loads in lungs and no mortality. While these studies emphasize the importance of ESX-3 in the virulence of *Mab* S, its contribution to the pathogenesis of *Mab* R *in vivo* remains undefined. Here, we exploited an *Mab* R mutant lacking *eccC3* using the zebrafish model, particularly suited to describe the chronology and pathophysiological signs of *Mab* R infection. Our results clearly show that R Δ*eccC3* was highly attenuated in larvae, as evidenced by the enhanced larvae survival, the reduced bacterial burden, cord number, and limited proportion of abscesses, while functional complementation of *eccC3* partially restored the phenotypes to wild-type levels. The phenotypes were corroborated with whole larvae imaging, showing lower bacterial foci in R Δ*eccC3*, as compared to the parental R strain. Moreover, infection with R Δ*eccC3* was associated with a lower pro-inflammatory response, as showcased by the reduced production of *il1b* and *tnfa* transcripts. Together, these findings demonstrate that ESX-3 is a primary driver of *Mab* R pathogenicity.

**IMPORTANCE:**

*Mycobacterium abscessus* (*Mab*) is currently recognized as an extremely difficult-to-manage pathogen in patients with cystic fibrosis. The rough (R) morphotype of *Mab* is associated with chronic and more aggressive infections in patients than the smooth (S) morphotype. However, the mechanisms of pathogenesis of *Mab* R remain largely unexplored. In this study, we investigated the infection capacity and virulence of a *Mab* R mutant lacking a functional ESX-3 secretion system using the zebrafish model. The mutant was severely impaired in its ability to replicate in the larvae, and this was correlated with a significant increase in larvae survival, highlighting the critical role of ESX-3 in *Mab* R virulence. Overall, this emphasizes ESX-3 as an attractive drug target for future drug developments against *Mab* R lung diseases.

## OBSERVATION

**Fig 1:**
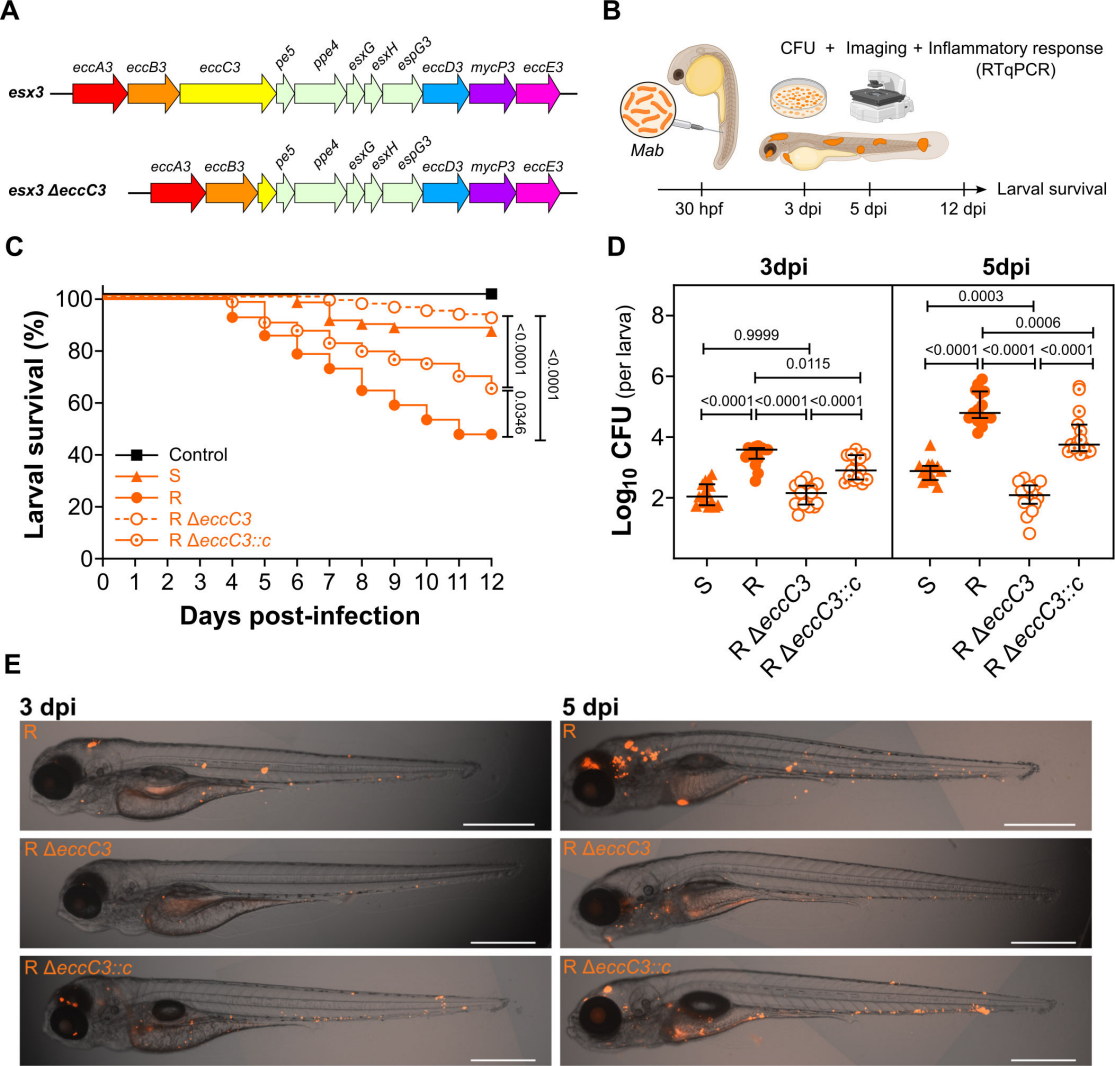
Virulence of the *M. abscessus* ESX-3 mutant in zebrafish larvae. (**A**) Genetic organization of the *esx-3* locus in *Mab* WT and Δ*eccC3*. (**B**) Schematic overview of the *in vivo* experiments. (**C**) Survival of infected larvae up to 12 days post-infection (dpi). The graph shows results from three pooled independent experiments with at least *n*=24 larvae per strain per experiment. Larvae were considered dead in the absence of a heartbeat. Statistical analysis was performed using the Log-rank (Mantel-Cox) test. (**D**) Bacterial burden of infected larvae at 3 dpi and 5 dpi. The graph shows results from three pooled independent experiments with *n*=5 larvae per strain per timepoint per experiment. CFU counts were Log_10_ transformed. Statistical analysis was performed using ordinary one-way ANOVA with Tukey’s multiple comparisons test, after verifying the normality of the data. (**E**) Representative images of infected larvae with R, R Δ*eccC3,* and R Δ*eccC3::c* at 3 dpi (left panels) and 5 dpi (right panels). Images were acquired in Widefield mode (Axiocam 712) with a TL LED lamp for Brightfield and an LED-Module 590 nm for mScarlet and tdTomato. Scale bar=500 µm.

Type VII Secretion Systems (T7SS), also known as the ESAT-6 secretion system (ESX) (1), allow for the secretion of a broad range of effectors across the cytoplasmic membrane. The number of ESX loci varies greatly from one mycobacterial species to another. *Mycobacterium abscessus (Mab),* a nontuberculous mycobacterium (NTM) that contributes significantly to the global rise of NTM infections and responsible for severe lung diseases in cystic fibrosis patients (2, 3), possesses only two ESX systems, ESX-3 and ESX-4 (4, 5). The ESX machineries share conserved core components (EccA, EccB, EccC, EccD, EccE, and the protease MycP) that form a channel across the inner membrane to facilitate substrate transport (1). In addition to these core component genes, the *esx-3* locus also encodes for small-secreted proteins with a conserved WXG motif (EsxG/EsxH), two *pe*/*ppe* genes (PE5 and PPE4), and *espG3* that encodes a chaperone binding-PE/PPE protein (Fig. 1A). Under standard culture conditions, *Mycobacterium tuberculosis* growth depends on ESX-3, while this is not the case for *Mab* (6). Recent studies have also revealed a functional link between ESX-3 and iron uptake in *Mab* and have shown that interfering with ESX-3 function results in the production of an unusual mycobactin siderophore (7). Biochemical assays showed that ESX-3 not only secretes EsxG/EsxH, PE5/PP4, but also other PE/PPE substrates (MAB_0046/MAB_0047) (8). We recently showed that the deletion of *eccC3* in *Mab* correlated with reduced bacterial internalization, phagosomal escape, and intracellular survival in THP-1 cells (8). In mice, infection with this mutant in the smooth (S) background resulted in increased survival and reduced bacterial loads in the lungs, indicating that ESX-3 drives *Mab* pathogenicity (8).

*Mab* changes its morphology and surface properties, which greatly influences its pathogenesis (2, 9). The synthesis of surface-associated glycopeptidolipids (GPL) typifies the S morphotype, whereas the lack of GPL synthesis and/or transport correlates with the emergence of a rough (R) morphotype associated with enhanced virulence (10, 11). Epidemiological studies suggested the greater impact of *Mab* R isolates in severe lung infections and chronic colonization of airways in patients (12–14). In addition, the R variant forms cords *in vitro* and also in infected zebrafish larvae (15–17), participating in immune evasion as these large bacterial structures are not efficiently internalized and destroyed by phagocytic cells (16). Herein, we investigated the contribution of ESX-3 in the pathogenesis of *Mab* R during infection in zebrafish larvae (16, 18). All *Mab* strains used in this study were derived from the CIP104536^T^ reference strain (19) (Table S1) and grown at 37°C in Middlebrook 7H9 broth (BD Difco) supplemented with 0.025% tyloxapol and 10% OADC enrichment (oleic acid, albumin, dextrose, and catalase), with antibiotics when required. The unmarked *eccC3* mutant in the R strain (R Δ*eccC3*) was designed to delete 3780 bp (94%) of the *eccC3* open reading frame (Fig. 1A) (8). Zebrafish (*Danio rerio*) were housed and handled in compliance with European Union guidelines for laboratory animal care, approved by the Direction Sanitaire et Vétérinaire de l’Hérault for the ZEFIX-CRBM zebrafish facility (Montpellier) under registration number C-34-172-39. Experimental protocols were approved by the “Ministère de l’Enseignement Supérieur, de la Recherche et de l’Innovation” under the reference APAFIS#24406-2020022815234677 V3. Golden mutant zebrafish (20) were raised under a 12/12 h light/dark cycle at the ZEFIX-CRBM zebrafish facility. Eggs were obtained by natural spawning and incubated at 28.5°C in Petri dishes containing E3 medium (5 mM NaCl, 0.17 mM KCl, 0.33 mM CaCl_2_, and 0.33 mM MgSO_4_). At 24 h post-fertilization (hpf), embryos were dechorionated enzymatically using 1 mg/mL pronase. To examine the role of ESX-3 in virulence, 30 hpf embryos were injected as described (21), with approximately 250 colony-forming units (CFU) of *Mab* S, *Mab* R, *Mab* R Δ*eccC3,* all expressing mScarlet, or complemented strain R Δ*eccC3::c* expressing tdTomato (Table S1). Injected embryos were randomly assigned for survival assays, bacterial burden, RT-qPCR of inflammatory cytokines, and imaging (Fig. 1B). Inoculum validation was performed by microinjecting a PBS drop and plating it on 7H10^OADC^. All statistical analysis was performed using PRISM 10.4.1 (GraphPad). While the R parental strain led to 48% of larval survival at 12 days post-infection (dpi), R Δ*eccC3* exhibited survival rates comparable to the S variant, with 92% and 89% survival, respectively (Fig. 1C). Complementation of R Δ*eccC3* partially restored virulence, enhancing survival to 67% at 12 dpi. To corroborate these results, we quantified bacterial burden at 3 and 5 dpi by counting CFUs in individual larva lysates, as previously described (21). Compared to the R parental strain, larvae infected with R Δ*eccC3* had significantly lower bacterial loads at 3 dpi and 5 dpi, similar to the S variant, while complementation of R Δ*eccC3* partially restored the observed phenotype to wild-type levels (Fig. 1D).

At 3 dpi and 5 dpi, larvae were anesthetized with 0.02% buffered MS222 and transferred individually in black 96-well plates with transparent bottoms. Larvae imaging was conducted using the Cell Discoverer 7 (Zeiss). The full 96-well plate was imaged using Tiles with the Plan-Apochromat 5×/0.35 objective and 0.5× Tubulense optovar. Images of full larvae were obtained after stitching and analyzed with ZEN 3.7 software. Imaging corroborated the CFU results, showing lower bacterial foci in R Δ*eccC3*, as compared to the parental R or the Δ*eccC3::c* strains (Fig. 1E).

**Fig 2:**
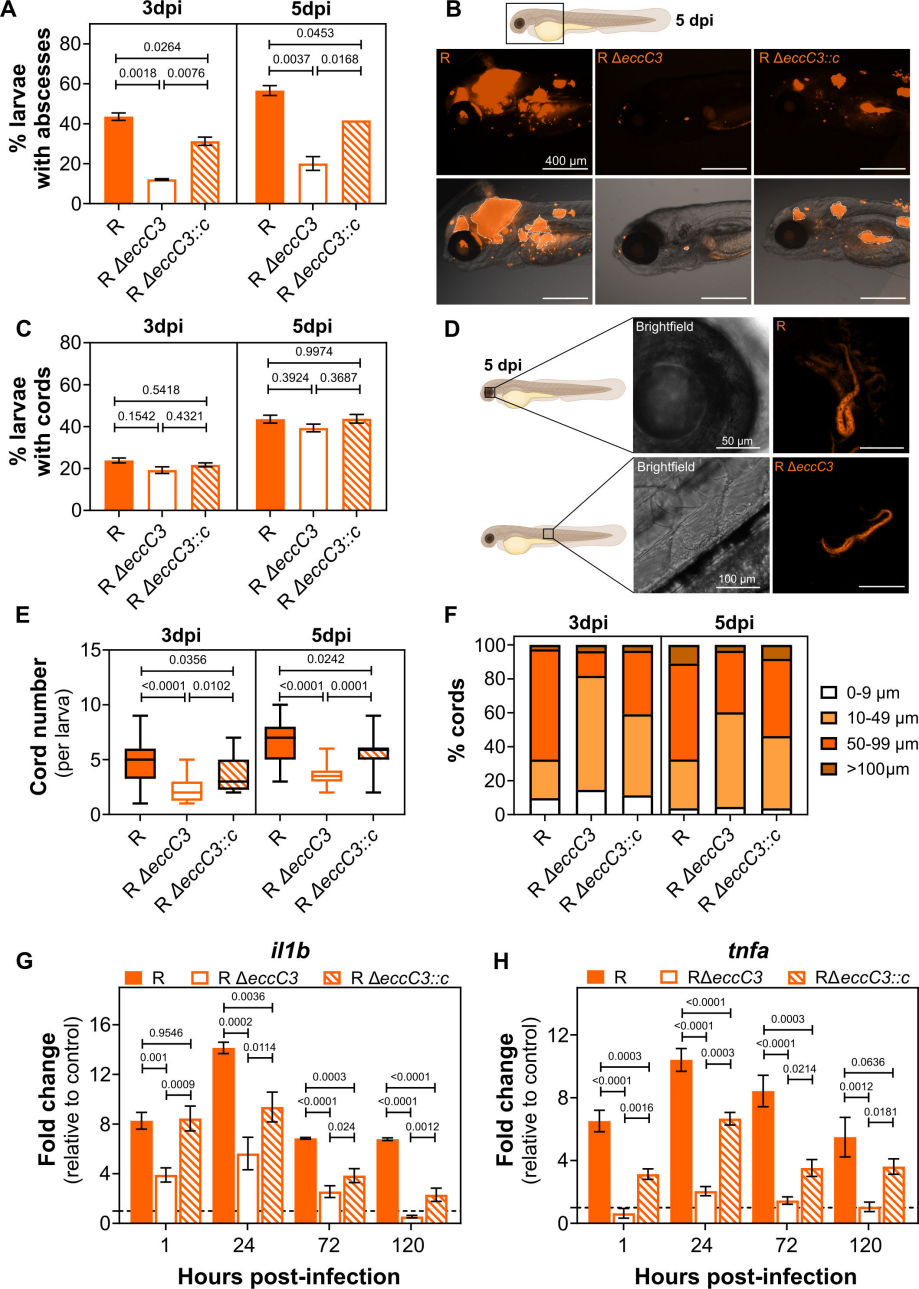
Pathophysiological indicators of infection in zebrafish larvae infected with the *Mab* ESX-3 mutant. (**A**) The percentage of larvae with abscesses after infection with R, R Δ*eccC3,* and R Δ*eccC3::c* at 3 days post-infection (dpi) and 5 dpi, and (**B**) representative images. Images were acquired in Widefield mode (Axiocam 712) with a TL LED lamp for brightfield and an LED-Module 590 nm for mScarlet and tdTomato. (**C**) The percentage of larvae with cords after infection with R, R Δ*eccC3,* and R Δ*eccC3::c* at 3 dpi and 5 dpi, and (**D**) representative images. Images were acquired in confocal mode with the 561 laser. Images are orthogonal projections of z-stacks. (**E**) the number of cords per larva and (**F**) the size distribution of cords at 3 dpi and 5 dpi. (**A, C, E, and F**) Data represent results from three pooled independent experiments, with an average of 24 larvae per condition per experiment. Relative *il1b* (**G**) and *tnfa* (**H**) gene expression measured by RT-qPCR at 1 h post-infection (hpi), 24 hpi, 72 hpi, and 120 hpi. PBS-injected embryos/larvae were used as controls. Data are from three pooled independent experiments, with 20 embryos/larvae per condition per experiment. (**A, C, E, G, and H**) Statistical analysis was performed per timepoint using ordinary one-way ANOVA with Tukey’s multiple comparisons test and after verifying the normality of the data.

Infection with *Mab* R is characterized by the formation of cords and abscesses, which are markers of disease severity (16). Using whole-larvae imaging, we quantified abscess and cord formation at 3 and 5 dpi (Fig. 2A; Supplemental material). Lower bacterial loads in R Δ*eccC3* correlated with fewer and smaller abscesses at both time points (Fig. 2A and B), and complementation partially rescued the proportion of larvae with abscesses. No difference in bacterial cording was observed between R and R Δ*eccC3*, suggesting that *eccC3* deletion does not impair cord formation (Fig. 2C and D). However, the number of cords per larva was reduced (Fig. 2E), and the size distribution of cords was smaller in larvae infected with the mutant (Fig. 2F), likely due to the reduced bacterial burden. Finally, we analyzed by quantitative RT-PCR the expression of the pro-inflammatory cytokines *il1b* and *tnfa,* known to be highly expressed upon *Mab* infection (22). RNA was extracted from 20 pooled embryos/larvae per condition per experiment using the RNeasy kit (QIAGEN), followed by reverse transcription and quantitative PCR with the Luna Universal One-Step RT-qPCR Kit (New England Biolabs) on a CFX opus real-time 384 system (BioRad) using 50 ng of RNA. Data were normalized to the housekeeping gene *ppial2* and analyzed using the ΔΔ*CT* method relative to control larvae. Primers used are listed in Table S2. Levels of *il1b* and *tnfa* were much lower in larvae infected with R Δ*eccC3* compared to the parental R strain, even at 1 hour post-infection (hpi) when bacterial loads are similar, while complementation partially restored the response (Fig. 2G and H), suggesting that a functional ESX-3 secretion system is required for an acute pro-inflammatory infection.

In summary, our study demonstrates that while dispensable *in vitro*, the loss of ESX-3 is necessary for *in vivo* growth of *Mab* R, resulting in an extremely attenuated phenotype in zebrafish larvae in the sole context of innate immunity. Future studies should discriminate whether this reduced pathogenesis is linked to the role of ESX-3 in iron homeostasis or in the secretion of substrates involved in the interaction with the innate immune cells. Together with previous work in mice (8), these results underscore ESX-3 as an attractive drug target to control the difficult-to-manage *Mab* infections.

## Data Availability

All data generated in this study are available upon request.

## SUPPLEMENTAL MATERIAL

Supplemental material spectrum.01949-25-s0001.pdf Supplemental methods; Tables S1 and S2.

ASM does not own the copyrights to Supplemental Material that may be linked to, or accessed through, an article. The authors have granted ASM a non-exclusive, world-wide license to publish the Supplemental Material files. Please contact the corresponding author directly for reuse.

## References

[1] Bitter W, Houben ENG, Bottai D, Brodin P, Brown EJ, Cox JS, Derbyshire K, Fortune SM, Gao L-Y, Liu J, Gey van Pittius NC, Pym AS, Rubin EJ, Sherman DR, Cole ST, Brosch R. 2009. Systematic genetic nomenclature for type VII secretion systems. PLoS Pathog 5:e1000507. doi:10.1371/journal.ppat.1000507 19876390 PMC2763215

[2] Johansen MD, Herrmann J-L, Kremer L. 2020. Non-tuberculous mycobacteria and the rise of Mycobacterium abscessus. Nat Rev Microbiol 18:392–407. doi:10.1038/s41579-020-0331-1 32086501

[3] Griffith DE, Aksamit T, Brown-Elliott BA, Catanzaro A, Daley C, Gordin F, Holland SM, Horsburgh R, Huitt G, Iademarco MF, Iseman M, Olivier K, et al.. 2007. ATS mycobacterial diseases subcommittee, American thoracic society, infectious disease society of America. Am J Respir Crit Care Med 175:367–416. doi:10.1164/rccm.200604-571ST 17277290

[4] Lagune M, Petit C, Sotomayor FV, Johansen MD, Beckham KSH, Ritter C, Girard-Misguich F, Wilmanns M, Kremer L, Maurer FP, Herrmann J-L. 2021. Conserved and specialized functions of type VII secretion systems in non-tuberculous mycobacteria. Microbiology (Reading) 167:001054. doi:10.1099/mic.0.001054 34224347

[5] Laencina L, Dubois V, Le Moigne V, Viljoen A, Majlessi L, Pritchard J, Bernut A, Piel L, Roux A-L, Gaillard J-L, Lombard B, Loew D, et al.. 2018. Identification of genes required for Mycobacterium abscessus growth in vivo with a prominent role of the ESX-4 locus. Proc Natl Acad Sci USA 115:E1002–E1011. doi:10.1073/pnas.1713195115 29343644 PMC5798338

[6] Rifat D, Chen L, Kreiswirth BN, Nuermberger EL. 2021. Genome-wide essentiality analysis of Mycobacterium abscessus by saturated transposon mutagenesis and deep sequencing. mBio 12:e0104921. doi:10.1128/mBio.01049-21 34126767 PMC8262987

[7] Bythrow GV, Farhat MF, Levendosky K, Mohandas P, Germain GA, Yoo B, Quadri LEN. 2022. Mycobacterium abscessus mutants with a compromised functional link between the type VII ESX-3 system and an iron uptake mechanism reliant on an unusual mycobactin siderophore. Pathogens 11:953. doi:10.3390/pathogens11090953 36145386 PMC9505556

[8] Daher W, Le Moigne V, Tasrini Y, Parmar S, Sexton DL, Aguilera-Correa JJ, Berdal V, Tocheva EI, Herrmann J-L, Kremer L. 2025. Deletion of ESX-3 and ESX-4 secretion systems in Mycobacterium abscessus results in highly impaired pathogenicity. Commun Biol 8:166. doi:10.1038/s42003-025-07572-4 39900631 PMC11791044

[9] Roux A-L, Viljoen A, Bah A, Simeone R, Bernut A, Laencina L, Deramaudt T, Rottman M, Gaillard J-L, Majlessi L, Brosch R, Girard-Misguich F, Vergne I, de Chastellier C, Kremer L, Herrmann J-L. 2016. The distinct fate of smooth and rough Mycobacterium abscessus variants inside macrophages. Open Biol 6:160185. doi:10.1098/rsob.160185 27906132 PMC5133439

[10] Howard ST, Rhoades E, Recht J, Pang X, Alsup A, Kolter R, Lyons CR, Byrd TF. 2006. Spontaneous reversion of Mycobacterium abscessus from a smooth to a rough morphotype is associated with reduced expression of glycopeptidolipid and reacquisition of an invasive phenotype. Microbiology (Reading) 152:1581–1590. doi:10.1099/mic.0.28625-0 16735722

[11] Pawlik A, Garnier G, Orgeur M, Tong P, Lohan A, Le Chevalier F, Sapriel G, Roux A-L, Conlon K, Honoré N, Dillies M-A, Ma L, Bouchier C, Coppée J-Y, Gaillard J-L, Gordon SV, Loftus B, Brosch R, Herrmann JL. 2013. Identification and characterization of the genetic changes responsible for the characteristic smooth-to-rough morphotype alterations of clinically persistent Mycobacterium abscessus. Mol Microbiol 90:612–629. doi:10.1111/mmi.12387 23998761

[12] Jönsson BE, Gilljam M, Lindblad A, Ridell M, Wold AE, Welinder-Olsson C. 2007. Molecular epidemiology of Mycobacterium abscessus, with focus on cystic fibrosis. J Clin Microbiol 45:1497–1504. doi:10.1128/JCM.02592-06 17376883 PMC1865885

[13] Catherinot E, Roux A-L, Macheras E, Hubert D, Matmar M, Dannhoffer L, Chinet T, Morand P, Poyart C, Heym B, Rottman M, Gaillard J-L, Herrmann J-L. 2009. Acute respiratory failure involving an R variant of Mycobacterium abscessus. J Clin Microbiol 47:271–274. doi:10.1128/JCM.01478-08 19020061 PMC2620830

[14] Hedin W, Fröberg G, Fredman K, Chryssanthou E, Selmeryd I, Gillman A, Orsini L, Runold M, Jönsson B, Schön T, Davies Forsman L. 2023. A rough colony morphology of Mycobacterium abscessus is associated with cavitary pulmonary disease and poor clinical outcome. J Infect Dis 227:820–827. doi:10.1093/infdis/jiad007 36637124 PMC10043986

[15] Sánchez-Chardi A, Olivares F, Byrd TF, Julián E, Brambilla C, Luquin M. 2011. Demonstration of cord formation by rough Mycobacterium abscessus variants: implications for the clinical microbiology laboratory. J Clin Microbiol 49:2293–2295. doi:10.1128/JCM.02322-10 21490192 PMC3122772

[16] Bernut A, Herrmann J-L, Kissa K, Dubremetz J-F, Gaillard J-L, Lutfalla G, Kremer L. 2014. Mycobacterium abscessus cording prevents phagocytosis and promotes abscess formation. Proc Natl Acad Sci USA 111:E943–52. doi:10.1073/pnas.1321390111 24567393 PMC3956181

[17] Bernut Audrey, Viljoen A, Dupont C, Sapriel G, Blaise M, Bouchier C, Brosch R, de Chastellier C, Herrmann J-L, Kremer L. 2016. Insights into the smooth-to-rough transitioning in Mycobacterium bolletii unravels a functional Tyr residue conserved in all mycobacterial MmpL family members. Mol Microbiol 99:866–883. doi:10.1111/mmi.13283 26585558

[18] Johansen MD, Spaink HP, Oehlers SH, Kremer L. 2024. Modeling nontuberculous mycobacterial infections in zebrafish. Trends Microbiol 32:663–677. doi:10.1016/j.tim.2023.11.011 38135617

[19] Ripoll F, Pasek S, Schenowitz C, Dossat C, Barbe V, Rottman M, Macheras E, Heym B, Herrmann J-L, Daffé M, Brosch R, Risler J-L, Gaillard J-L. 2009. Non mycobacterial virulence genes in the genome of the emerging pathogen Mycobacterium abscessus. PLoS One 4:e5660. doi:10.1371/journal.pone.0005660 19543527 PMC2694998

[20] Lamason RL, Mohideen M-A, Mest JR, Wong AC, Norton HL, Aros MC, Jurynec MJ, Mao X, Humphreville VR, Humbert JE, Sinha S, Moore JL, et al.. 2005. SLC24A5, a putative cation exchanger, affects pigmentation in zebrafish and humans. Science 310:1782–1786. doi:10.1126/science.1116238 16357253

[21] Boudehen Y-M, Tasrini Y, Aguilera-Correa JJ, Alcaraz M, Kremer L. 2023. Silencing essential gene expression in Mycobacterium abscessus during infection. Microbiol Spectr 11:e0283623. doi:10.1128/spectrum.02836-23 37831478 PMC10714871

[22] Bernut A, Nguyen-Chi M, Halloum I, Herrmann J-L, Lutfalla G, Kremer L. 2016. Mycobacterium abscessus-induced granuloma formation is strictly dependent on TNF signaling and neutrophil trafficking. PLoS Pathog 12:e1005986. doi:10.1371/journal.ppat.1005986 27806130 PMC5091842

