# Supplementary material for: EccC3 is essential for pathogenesis of rough Mycobacterium abscessus in zebrafish
Source: Microbiol Spectr. 2025 Sep 29;13(11):e01949-25. doi: 10.1128/spectrum.01949-25 (PMC12584687; doi:10.1128/spectrum.01949-25)
Supplement: Supplemental material — Supplemental methods; Tables S1 and S2. [file spectrum.01949-25-s0001.pdf]

Supplemental material

EccC3 is essential for pathogenesis of rough *Mycobacterium abscessus* in zebrafish

Yara Tasrini[1], Wassim Daher[1,2], and Laurent Kremer[1,2*]

[1]Centre National de la Recherche Scientifique UMR 9004, Institut de Recherche en Infectiologie de Montpellier (IRIM), Université de Montpellier, 1919 route de Mende, 34293, Montpellier, France.
[2]INSERM, IRIM, 34293 Montpellier, France.

**Corresponding author: Laurent Kremer;* Tel: (+33) 4 34 35 94 47; *E-mail:* laurent.kremer@irim.cnrs.fr

Running title: *M. abscessus pathogenesis in zebrafish relies on ESX-3*

Keywords: *Mycobacterium abscessus, ESX-3 secretion system, pathogenesis, infection, zebrafish.*

Supplementary Methods

Quantification of cords and abscesses in zebrafish larvae.

To quantify cords and abscesses, anesthetized larvae were imaged at high magnification (≥ 20x). Cords were identified as elongated, filamentous structures with uniform bacterial fluorescence, whereas abscesses were identified as mostly circular, compact structures with diffuse fluorescence and a necrotic center visible in bightfield as an opacification spot. If a cord or abscess was detected in a larva, the larva was scored as “positive”, resulting in the percentage of larvae with cords or abscesses. The number of cords per larva was counted, and cord size was measured using ZEN 3.7 software.

Supplementary Tables

Table S1. Strains used in this study.

Strain	Description	Reference
M. abscessus Smooth	CIP104536^T (smooth morphotype), used as the parental strain	Laboratoire National des Mycobactéries
M. abscessus Smooth (*Mab* S) mScarlet	CIP104536^T Smooth reference strain transformed with the integrative pMV306-P_{left}**mScarlet*-Kan^R	(1)
M. abscessus Rough (*Mab* R) mScarlet	CIP104536^T Rough reference strain transformed with the integrative pMV306-P_{left}**mScarlet*-Kan^R	(1)
Mab R Δ*eccC3 mScarlet*	*Mab* R Δ*eccC3* mutant transformed with the integrative pMV306-P_{left}**mScarlet*-Kan^R	(2)
Mab R Δ*eccC3::c tdTomato*	*Mab* R Δ*eccC3* complemented with the pMV306:*eccC3_HA* and transformed with the episomal pTEC27-tdTomato-Hyg^R	(2)

Reference

1. Pichler V, Dalkilic L, Shoaib G, Shapira T, Rankine-Wilson L, Boudehen Y-M, Chao JD, Sexton D, Prieto M, Quon BS, Tocheva EI, Kremer L, Hsiao W, Av-Gay Y. 2024. The diversity of clinical *Mycobacterium abscessus* isolates in morphology, glycopeptidolipids and infection rates in a macrophage model. J Med Microbiol 73**:** 001869

2. Daher W, Le Moigne V, Tasrini Y, Parmar S, Sexton DL, Aguilera-Correa JJ, Berdal V, Tocheva EI, Herrmann J-L, Kremer L. 2025. Deletion of ESX-3 and ESX-4 secretion systems in *Mycobacterium abscessus* results in highly impaired pathogenicity. Commun Biol 8:166.

Table S2. Primers used in this study.

Name	Sequence
qPCR-ppial2-For	5’-ACACTGAAACACGGAGGCAAAG-3’
qPCR-ppial2-Rev	5’-CATCCACAACCTTCCCGAACAC-3’
qPCR-il1b-For	5’-CGCTCCACATCTCGTACTCA-3’
qPCR-il1b-Rev	5’-ATACGCGGTGCTGATAAACC-3’
qPCR-tnfa-For	5’-GCGCTTTTCTGAATCCTACG-3’
qPCR-tnfa-Rev	5’- TGCCCAGTCTGTCTCCTTCT-3’
